# Insights into the Molecular Mechanisms of the Anti-Atherogenic Actions of Flavonoids in Normal and Obese Mice

**DOI:** 10.1371/journal.pone.0024634

**Published:** 2011-10-10

**Authors:** Elena V. Shabrova, Olga Tarnopolsky, Ajay P. Singh, Jorge Plutzky, Nicholi Vorsa, Loredana Quadro

**Affiliations:** 1 Department of Plant Biology and Pathology, Rutgers, The State University of New Jersey, New Brunswick, New Jersey, United States of America; 2 Cardiovascular Division, Department of Medicine, Brigham and Women's Hospital and Harvard Medical School, Cambridge, Massachusetts, United States of America; 3 PE Marucci Center, Rutgers, The State University of New Jersey, Chatsworth, New Jersey, United States of America; 4 Food Science Department and Rutgers Center for Lipid Research, Rutgers, The State University of New Jersey, New Brunswick, New Jersey, United States of America; Sapienza University of Rome, Italy

## Abstract

Obesity is a major and independent risk factor for cardiovascular disease and it is strongly associated with the development of dyslipidemia, insulin resistance and type 2 diabetes. Flavonoids, a diverse group of polyphenol compounds of plant origin widely distributed in human diet, have been reported to have numerous health benefits, although the mechanisms underlying these effects have remained obscure. We analyzed the effects of chronic dietary supplementation with flavonoids extracted from cranberry (FLS) in normal and obese C57/BL6 mice compared to mice maintained on the same diets lacking FLS. Obese mice supplemented with flavonoids showed an amelioration of insulin resistance and plasma lipid profile, and a reduction of visceral fat mass. We provide evidence that the adiponectin-AMPK pathway is the main mediator of the improvement of these metabolic disorders. In contrast, the reduced plasma atherogenic cholesterol observed in normal mice under FLS seems to be due to a downregulation of the hepatic cholesterol synthesis pathway. Overall, we demonstrate for the first time that the molecular mechanisms underlying the beneficial effects of flavonoids are determined by the metabolic state.

## Introduction

Flavonoids have been consumed in various forms throughout the course of human existence. Over the last 20 years, these diverse polyphenol compounds of plant origin have received considerable attention due to their potential benefits to human health [1]. Population studies have revealed an inverse correlation between flavonoid intake from regular food and incidence of several chronic diseases, including cardiovascular diseases (CVD) [2]. Despite this, how flavonoids exert their effects has remained poorly defined. A major focus has been on the antioxidant properties of flavonoids, although they likely exert their effects mainly through the modulation of signaling pathways and gene expression [3]–[5]. A deeper understanding of the mechanisms of flavonoid action *in vivo* is required to evaluate and better utilize their potential for the treatment and prevention of human diseases.

Obesity is now recognized as a major contributor to multiple health issues. Obesity has been strongly linked to dyslipidemia, including elevated triglycerides (TG) and low density lipoproteins (LDL), and low high density lipoproteins (HDL). Increased central adiposity is also associated with microalbuminuria, inflammation, hypertension, insulin resistance, and the risk for future diabetes. Given these effects, obesity has also been implicated in the risk of CVD [6]. These multiple metabolic abnormalities may derive from increased adiposity in visceral fat depots as well as in abnormal settings, such as liver and skeletal muscle. In addition to energy storage, white adipose tissue is now understood as a critical endocrine organ secreting adipokines, a large group of adipocyte-derived signaling molecules diverse in their structure and function [7]. Although many adipokines are thought to adversely affect metabolism and promote inflammation, others may have protective actions. Adiponectin is an adipokine with anti-diabetic and anti-atherogenic effects [8]. Adiponectin administration reverses insulin resistance in lipoatrophic diabetic mice as well as in obese and type 2 diabetic mouse models [8].

Flavonoids present in cranberry have shown many beneficial effects, including the antidiabetic [4], [9], antihypertensive [10], and cardioprotective [9] effects of quercetin; antiobesity effects of anthocyanins [11]; as well as the contribution of proanthocyanidins to the so-called “French paradox” [12]. As such, we hypothesized that dietary supplementation with a cranberry extract enriched in flavonoids (FLS) would have direct effects on metabolic abnormalities associated with obesity and provide health benefits under a normal metabolic status. Therefore, we investigated the effects of FLS in wild-type mice fed either high-fat or low-fat diets (HFD or LFD). Indeed, obese mice supplemented with flavonoids showed an amelioration of insulin resistance and plasma lipid profile, and a reduction of visceral fat mass. We provide evidence that these effects might be mediated by the activation of the adiponectin-AMPK pathway. On the other hand, the reduced plasma atherogenic cholesterol observed in normal mice under FLS seems to be due to a downregulation of the hepatic cholesterol synthesis pathway. Therefore, our data suggest that the molecular mechanisms underlying the flavonoid effects depend upon the metabolic status.

## Materials and Methods

### *Cranberry extract*

A flavonoid extract was prepared from ‘90MX’ powder (Ocean Spray Cranberries, Inc. Lakeville-Middleboro, MA, USA) as previously described [13]. The composition of flavonoid extract, as shown in Table S1, was determined by HPLC analysis [13] .

### Animals and diets

Forty C57BL/6 male mice at 5 weeks of age were purchased from The Jackson Laboratory (Bar Harbor, ME, USA) and maintained on a standard chow diet for one week prior to the beginning of the study. Mice were maintained in a temperature-controlled facility (25°C) with a 12∶12 light/dark cycle, and given both diet and water on an *ad libitum* basis, except when fasting was required. At 6 weeks of age, mice were randomly assigned to two groups and maintained either on a low-fat diet (LFD; 10% energy from fat; Table S2) or on a high-fat diet (HFD; 60% energy from fat; Table S2) for 8 additional weeks (i.e. until 14 weeks of age). We chose this experimental protocol to induce obesity based on previously published reports [14], [15]. Next, mice were maintained either on the same dietary regimen (LFD or HFD) or on the same diet supplemented with 2% of flavonoid extract (LFDC or HFDC) for 10 additional weeks (i.e. until 24 weeks of age). Extract was added to the food in substitution of fiber and mixed to homogeneity during the manufacturing of the diets (TestDiet, Richmond, IN, USA). We chose the extract concentration based on pilot experiments performed with different concentrations dissolved in water or mixed with food. 2% of flavonoid extract was the maximum concentration achieved that did not alter food intake and/or exhibit obvious negative side effects. The weight of the mice and food intake were recorded on a weekly basis. Body composition analysis (by PIXImus DEXA scan, [16]) and glucose tolerance test (GTT) were performed on subgroups of mice at 14 and 24 weeks of age. Subsequently, mice were fasted overnight (16 hrs) and sacrificed to collect serum and tissues. At the time of sacrifice, selected organs, including liver, muscle, visceral fat and mesenteric fat, were dissected and weighed. All tissue samples and serum were frozen immediately and kept at −80°C for further analysis. All animal experiments were conducted in accordance with the National Institutes of Health Guide for the Care and Use of Laboratory Animals [17] and were approved by the Rutgers University Institutional Committee on Animal Care (protocol no. 05-009).

### Serum parameters

Serum levels of non-esterified fatty acids (NEFA), triglycerides (TG), total and HDL cholesterol (T-C and HDL-C) were measured using the HR Series NEFA-HR(2), L-Type TG H, Cholesterol E, and HDL-Cholesterol E kits, respectively (Wako Chemicals, Richmond, VA, USA). Atherogenic cholesterol (non-HDL) was estimated by subtracting HDL-C from T-C [18]. Insulin, leptin, resistin, and adiponectin were measured using Ultra-sensitive Rat/mouse insulin assay (Crystal Chem, Downers Grove IL, USA), Mouse Leptin Immunoassay kit (R&D System, Minneapolis, MN, USA), Resistin (murine) EIA Kit (Cayman Chemical Company, Ann Arbor, MI, USA), and Adiponectin (Mouse) EIA kit (ALPCO, Salem, NH, USA).

### Tissue lipid concentration

Total lipids from liver and muscle samples were extracted according to the Folch extraction protocol [19]. The extracts dissolved in chloroform with 2% Triton X100 were evaporated under nitrogen and dissolved in water (for TG and FA) or isopropanol (for cholesterol), followed by measurement of TG, total cholesterol and FA using L-Type TG H, Cholesterol E, and HR Series NEFA-HR(2) kits, respectively (Wako Chemicals, Richmond, VA, USA) [20].

### Western blot analysis

Monoclonal rabbit anti-mouse AMPKα and Phospho-AMPKα (Thr172) antibodies (Cell Signaling Technology, Danver, MA, USA), goat anti-mouse LDL receptor (LDLR) (R&D Systems, Inc., Minneapolis, MN, USA), rat monoclonal anti-human/mouse (specific for full-length protein) and monoclonal anti-mouse Adiponectin/Acr30 antibodies (for full-length and truncated proteins) (R&D System Inc., Minneapolis, MN, USA), rabbit polyclonal anti-β-tubulin antibody (IMGENEY, San Diego, CA, USA), anti-Tim23 (BD Biosciences, San Jose, CA, USA) were used as primary antibodies. Albumin and tubulin were used as loading controls. Albumin was detected upon staining of the membranes with Ponceau solution. For adiponectin multimers, serum samples (1 µl) were prepared in non-reducing loading buffer [21] and separated in 8-15% gradient SDS-PAGE run at +4°C and low voltage (20–40 volts). The quantification of the membranes was completed by densitometry analysis with Quantity One Program (Bio-Rad Laboratories, Hercules, CA, USA). Reference samples were loaded on each gel to allow normalization between gels. Western blot analysis was repeated several times (3 to 7) and results are shown as the average of signals obtained from the different gels for each sample.

### Total RNA extraction and real-time RT-PCR analysis

RNA samples from adipose, muscle, and liver tissues were extracted using RNeasy Lipid Tissue Mini Kit; RNeasy Fibrous Tissue Mini Kit, (QIAGEN, Valencia, CA, USA); and RNA Bee reagent (Tel-test Inc, TX, USA), respectively, followed by DNase I treatment (Roche Diagnostics, IN). Real time RT-PCR protocol has been described previously [16]. Cyclophilin A and β-actin were used as housekeeping genes. Primer sequences are shown in Table S3.

### Statistical analysis

Data are expressed as mean±SE. LFD group was used as a reference for quantitative analysis of mRNA and protein levels. Mean results were compared between supplemented and non-supplemented groups using a 2-tailed unpaired *t*-test with a threshold for significance at 0.05.

### Homeostatic model assessment (HOMA)

HOMA was calculated according to [22].

## Results

### FLS ameliorates metabolic abnormalities associated with obesity

#### Body and tissue weights and composition

**Figure 1:**
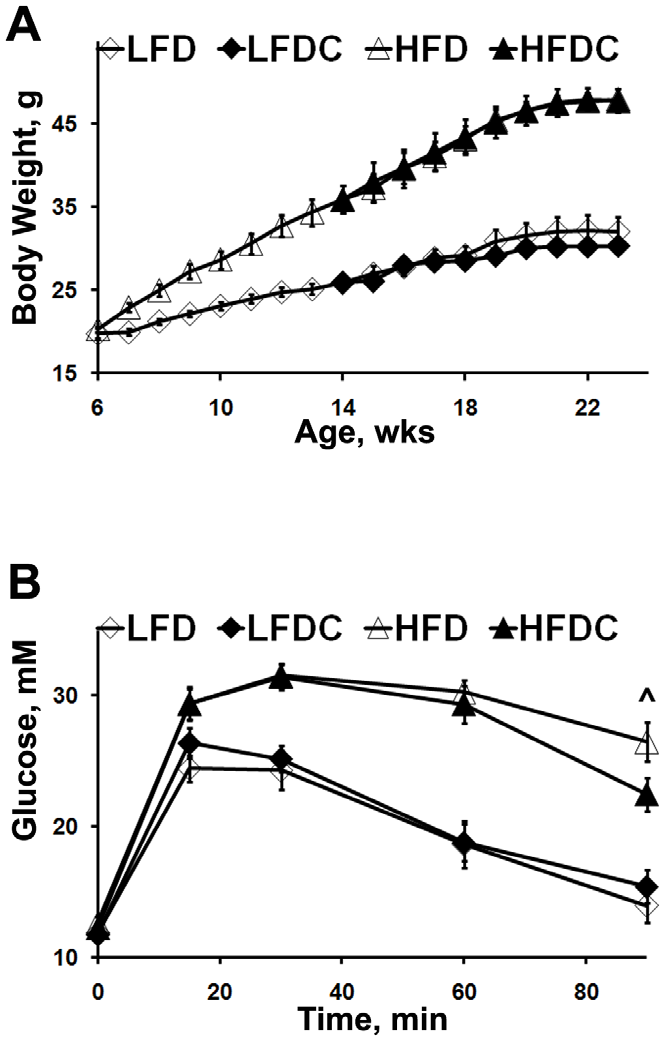
Body weight changes and Glucose Tolerance Test (GTT) in mice on different dietary regimens. A, mouse body weight was measured weekly from 6 to 24 weeks of age. n = 12 mice in each group at 6–14 wks of age; n = 8 mice in each group at 14–24 wks of age. B, GTT in mice at 24 wks of age. Glucose (1.5 g/ kg body weight) was injected intraperitoneally after overnight fasting (16 hours). Plasma glucose was measured using OneTouch Ultra Glucose Meter (LifeScan) at 0, 15, 30, 60, and 90 min after injection. Data are expressed as means±SE. ∧P = 0.06 HFDC vs. HFD, n = 6–8 mice per group.

We started FLS after obesity was induced in the high-fat fed group (Figure 1A). Specifically, after 8 weeks on HFD (i.e. at 14 weeks of age), a subgroup of mice was tested to confirm dietary effects. Indeed, mice fed HFD exhibited elevated fasting serum levels of insulin, glucose, leptin, TG, and impaired GTT compared to mice fed LFD (Table S4 and Figure S1). Throughout the study, mice steadily gained weight with significant increase on high-fat diets (Figure 1A). Although animals on LFDC weighed less than those on LFD, we did not observe any significant difference in body weight by the end of the study or any difference in total weight gain between supplemented and non-supplemented groups (data not shown). FLS also did not affect total adiposity. However, the analysis of organ weights revealed a significant reduction of visceral (gonadal) fat mass in HFDC mice (Table 1). Liver and muscle TG content was different between high- and low-fat fed groups, as expected (Table 1). In contrast, while no differences were detected in hepatic TG content among supplemented and non-supplemented groups, muscle TG (Table 1 and Figure S2) and FA (Figure S2) levels were lower in HFDC compared to the HFD group. Liver cholesterol levels were similar, regardless of the dietary regimen (Table 1).

**Table 1:** Body composition, tissue weight, tissue lipid content, and fasting serum parameters of mice at 24 weeks of age.

	LFD	LFDC	HFD	HFDC
Body composition				
Body weight, g	32±2	30±1	47±1	47±1
Lean mass^&^, g	23±2	21±0.5	23±3	23±1
Total fat^&^, %	32±7	33±6	54±3	54±2
Bone mineral density^#^, mg/cm^2^	50±2	50.5±0.2	43±4	47±1
Tissue weights and lipid content				
Visceral fat, g	1.2±0.7	0.95±0.4	2.4±0.4	1.9*±0.4
Visceral fat, %	3.1±0.6	3.1±0.4	5.4±0.3	4.1*±0.4
Mesenteric fat, g	0.30±0.11	0.32±0.05	1.40±0.11	1.42±0.09
Liver, g	1.10±0.08	1.01±0.02	1.9±0.2	1.8±0.2
Liver TG, mg/g tissue	16±2	17±1	47±5	41±4
Liver Cholesterol, mg/g tissue	2.4±0.2	2.6±0.1	2.7±0.1	3.0±0.2
Muscle TG, mg/g tissue	15±3	15±2	28 + 2	24*±1
Fasting serum parameters: Glucose metabolism				
Glucose, mM	11.6±0.5	11.7±0.5	12.6±0.5	12.2±0.4
GTT, glucose at 90 min, mM	13±1	15±1	27±1	23±1
Insulin, ng/mL	0.3±0.1	0.19±0.05	1.0±0.2	0.4*±0.2
HOMA	4.0±1.2	2.1±0.5	10±2	1.9*±0.8
Fasting serum parameters: Lipids				
TG, mg/dL	58±5	58±8	77±10	56±6
NEFA, mmol/L	0.88±0.05	0.91±0.05	1.04±0.14	1.15±0.06
T-C, mg/dL	140±7	123±4	193±12	183±9
HDL-C, mg/dL	90±5	90±3	101±6	118±6
(T-C)–(HDL-C), mg/dL	50±5	34*±2	92±9	65*±5
HDL-C/T-C, %	64±2	73*±2	53±2	65*±2
Fasting serum parameters: Adipokines				
Leptin, ng/mL	7±6	7±4	90±20	80±22
Resistin, ng/mL	26±2	34±5	35±5	22*±2
Adiponectin, µg/mL	53±6	74*±3	65±3	79*±4

*P<0.05 HFDC vs. HFD and LFDC vs. LFD, n = 6−8 animals per group except in ^&^, where n = 3. Data are expressed as mean±SE. Total fat (%) was calculated by using body weight and body fat mass obtained from the DEXA analysis. Visceral fat (%) was calculated by using direct weight measurements (i.e. by a scale) of mouse body weight at sacrifice and of dissected gonadal fat.

#### Serum parameters of glucose metabolism

Fasting serum glucose levels were similar among all groups (Table 1). GTT showed impaired glucose sensitivity in obese mice (HFD and HFDC), with a trend towards a reduction of glucose levels in the HFDC group, 90 min after glucose administration (Figure 1B). However, fasting insulin serum levels and HOMA values were significantly reduced in HFDC vs. HFD group (Table 1).

#### Serum parameters of lipid metabolism

Although there were no significant differences in fasting serum T-C and HDL-C levels, both supplemented groups showed a trend towards a decrease in T-C (Table 1). Of note, HFDC mice at 24 weeks of age displayed increased serum HDL-C levels and percentage compared to HFD mice at baseline, prior to FLS initiation (Table 1 and Table S4, P<0.05 and P<0.01, respectively), with no significant difference in T-C levels. Moreover, FLS significantly decreased atherogenic cholesterol (T-C *minus* HDL-C) and elevated the percentage of HDL-C (Table 1).

Fasting serum levels of TG showed a trend towards an increase in the HFD mice compared to all other groups, including the HFDC group (Table 1). NEFA levels were only significantly different between HFDC and HDF mice at baseline, (Table 1 and Table S4, P<0.05).

Overall these data show that FLS ameliorates insulin resistance and serum lipid profile, and also reduces visceral fat mass in obese mice.

#### Serum adipokines

**Figure 2:**
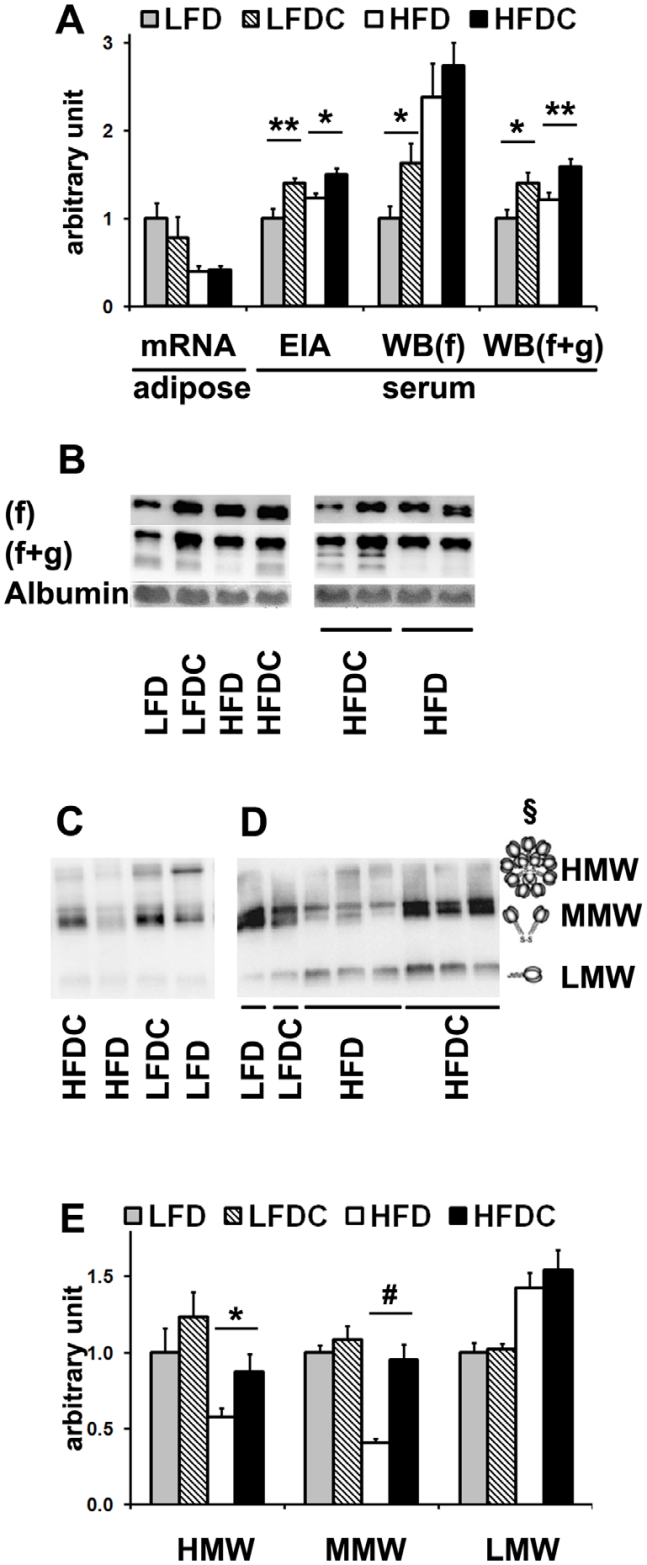
FLS increases adiponectin levels and restores multimers' adiponectin profile altered by high-fat feeding. A, quantitative analysis of adiponectin mRNA levels in visceral adipose tissue by real-time RT-PCR and adiponectin protein levels in serum by EIA or western blot (WB). B–D, representative western blots of serum samples. 0.5 µl (B) or 1 µl (C, D) of serum was subjected to 15% (B) or 8–15% (C, D) SDS-PAGE under reducing, heat-denaturing (B) or non-reducing, non-heat-denaturing (C, D) conditions at different voltage: 20–25 V (C), 40–45 V (D). Adiponectin was detected using antibodies specific for both globular (g) and full-length (f) adiponectin [(g+f), B–D)] or full length [(f), B)]. Albumin was detected by Ponceau staining (B) and used as a loading control for reducing conditions. E, quantitative analysis of adiponectin multimers in serum. Each serum sample was analyzed 2–4 times on different western blot membranes. HMW, high molecular weight; MMW, medium molecular weight; LMW, low molecular weight multimers. LFD group was used as a reference for quantification. Data are expressed as mean±SE. *P<0.05, **P<0.01, ^#^P<0.001 LFDC vs. LFD or HFDC vs. HFD; n = 7−8 in each group. §C, adopted from (21).

Given the reduced visceral adiposity seen with FLS, we next considered changes in specific adipokines that might be associated with this response. As expected [23], serum leptin levels were drastically elevated in obese mice, but with no differences upon FLS (Table 1). In contrast, serum levels of adiponectin were significantly elevated in FLS groups, both on low- and high-fat diets. Interestingly, adipose adiponectin mRNA levels (Figure 2A) were significantly reduced in HFD vs. LFD groups but not as a function of FLS.

To further investigate the pattern of circulating adiponectin we performed various western blot analyses (Figure 2). The antibody specific for the full-length protein revealed a doublet of bands corresponding to adiponectin isoforms generated upon posttranslational modifications (Figure 2B) [24]. This doublet showed a significant quantitative increase in LFDC vs. LFD with no significant difference between HFDC and HFD groups (Figure 2A). An antibody that detects full-length and truncated adiponectin proteins revealed minor lower molecular weight bands in addition to the above-mentioned doublet of full-length adiponectin (Figure 2B). The intensity of these minor bands was reduced in all HFD samples compared to all others. The doublet *plus* minor bands was quantitatively equivalent to EIA, showing significantly elevated adiponectin levels in both supplemented groups (Figure 2A).

Given adiponectin's presence in the circulation as trimers (low molecular weight, LMW), hexamers (medium molecular weight, MMW), and high molecular weight multimers (HMW) [25], [26], serum adiponectin profile was also analyzed by non-reducing SDS-PAGE followed by western blot analysis (Figure 2C and 2D). Consistent with previous reports [26], [27], our data also showed reduced levels of HMW (P<0.05, HFD vs. LFD and P<0.01, HFD vs. LFDC) and increased levels of LMW (P<0.01, HFD and HFDC vs. LFD and LFDC) multimers in the circulation of high-fat fed mice (Figure 2C and 2E). Moreover, MMW band was significantly reduced in the HFD group (Figure 2C and 2E). FLS restored the levels of both HMW and MMW complexes in obese mice without affecting LMW. Interestingly, HMW and MMW multimers were detected as multiple bands which possibly correspond to a variety of complexes assembled from adiponectin upon posttranslational modifications [24]. A variation of trimer numbers may also contribute to the complexity of the HMW area. Notably, gel filtration followed by Western blot analysis revealed a ladder of multiple bands for MMW and LMW fractions [28]. Lack of detection of multiple bands corresponding to trimer isoforms was probably due to poor resolution in the low molecular weight area. Notably, the non-reducing SDS-PAGE gels were run at low voltage, a condition that is known to improve the resolution of high molecular weight bands and reduce that of low molecular weight bands due to diffusion (Figure 2C and 2D). Hence, the significant decrease of LMW intensity (Figure 2C) when compared to that of LMW on gels run at higher voltage (Figure 2D). Overall, our data suggest that FLS restores the serum adiponectin profile altered by high-fat feeding.

A significant reduction in serum resistin levels indicates that this adipokine might be an additional factor underlying the improvement of insulin sensitivity in HFDC compared to HFD group (Table 1).

### Effect of FLS on cholesterol metabolism in liver

**Figure 3:**
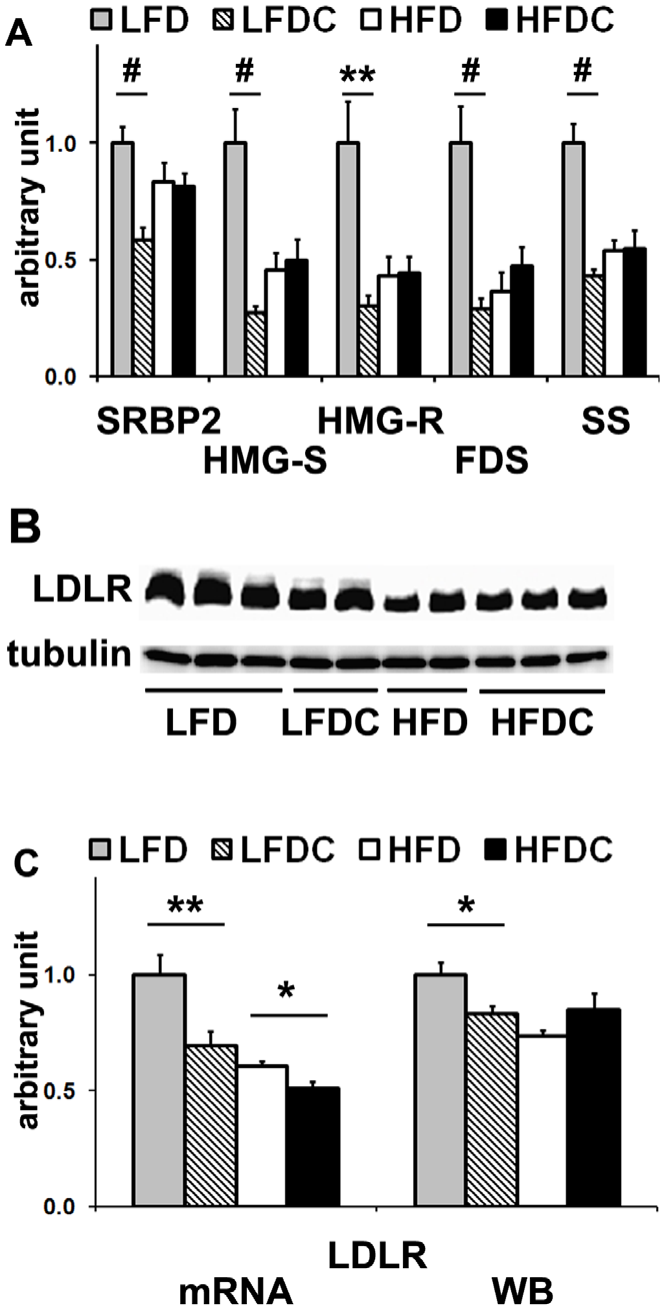
FLS downregulates expression of genes involved in hepatic cholesterol synthesis and hepatic LDL uptake in non-obese mice in fasting state. A, real-time RT-PCR analysis of genes involved in cholesterol synthesis and uptake in liver. B, representative western blot for low density lipoprotein receptor (LDLR). 70 µg of liver protein was subjected to 8% SDS-PAGE under reducing, heat-denaturing conditions. Tubulin was used as a loading control. Each sample was analyzed 2–4 times on different western blot membranes. LFD group was used as a reference for quantification. C, real-time RT-PCR analysis (mRNA) and quantification of western blot analysis (WB) for LDLR. Data are expressed as mean±SE. *P<0.05, **P<0.01, ^#^P<0.001 LFDC vs. LFD or HFDC vs. HFD; n = 7−8 in each group. Sterol-regulatory element binding protein 2 (SREBP2); 3-hydroxy-3-methylglutaryl-Co-A reductase (HMG-R); 3-hydroxy-3-methylglutaryl-CoA synthase (HMG-S); farnesyl diphosphate synthase (FDS); squalene synthase (SS).

Since circulating atherogenic cholesterol levels were reduced upon FLS, we measured hepatic mRNA levels of enzymes and transcription factors involved in cholesterol synthesis as well as those of receptors involved in cholesterol uptake. All the genes involved in cholesterol synthesis, with the exception of sterol regulatory element-binding protein-2 (SREBP2), as well as the gene encoding the low density lipoprotein receptor (LDLR) were downregulated in obese (HFD and HFDC) *vs.* normal mice (LFD) (Figure 3A and 3C). LDLR mRNA levels were also further reduced in HFDC vs. HFD-fed mice (Figure 3C). The pattern of LDLR protein levels mirrored that of mRNA levels in all the groups except HFDC. Indeed, LDLR in HFDC group showed a trend towards an increase compared to the HFD group and levels similar to LFD and LFDC groups (Figure 3B and 3C). Moreover, FLS significantly reduced mRNA levels of all analyzed genes in mice maintained on the low-fat diet (Figure 3A and 3C). No differences were observed among groups in hepatic mRNA levels of scavenger receptor class B type I (SRBI) and lipoprotein related protein (LRP) (data not shown).

These data suggest that the inhibition of hepatic cholesterol synthesis (and hence its secretion) could account for the reduced levels of circulating atherogenic cholesterol observed in LFDC group. The lack of differences in hepatic cholesterol content among groups (Table 1) emphasizes the complexity of the regulatory mechanisms that maintain homeostatic levels of cholesterol in mammalian cells [29].

### Effect of FLS on lipid metabolism in muscle

**Figure 4:**
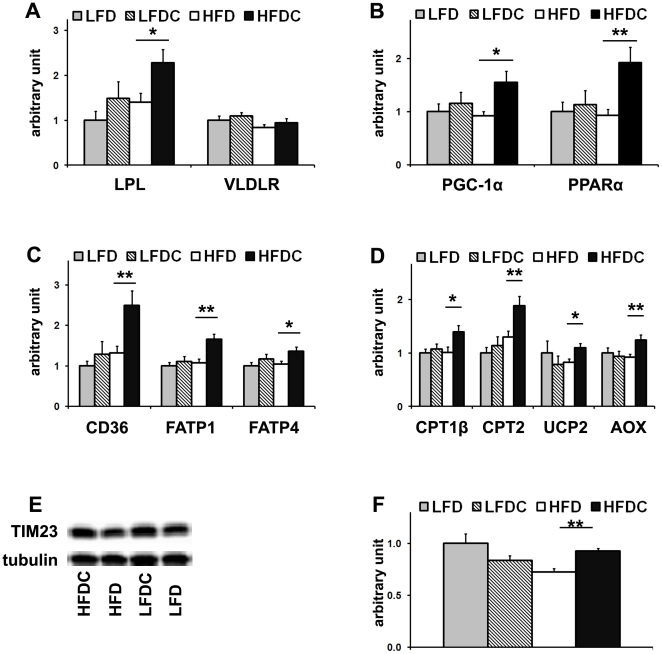
FLS upregulates expression of LPL and genes involved in FA utilization in muscle in obese mice. Real-time RT-PCR analysis of lipoprotein lipase (LPL) and very low density lipoprotein receptor (VLDLR) (A), genes involved in FA utilization in muscle: transcriptional regulation (B), free FA (FFA) uptake and activation (C), and FA oxidation (D). E, representative western blot for TIM23 (E). 50 µg of muscle protein was subjected to 15% SDS-PAGE under reducing, heat-denaturing conditions. Tubulin was used as a loading control. F, quantitative analysis of TIM23 in muscle. LFD group was used as a reference for quantification. Data are expressed as mean±SE. *P<0.05, **P<0.01, HFDC vs. HFD n = 8 in each group. Peroxisome proliferator-activated receptor α (PPARα); PPARγ coactivator-1α (PGC-1α); cluster of differentiation (CD36); fatty acid transport proteins 1 and 4 (FATP1 and FATP4); carnitine palmitoyltransferases 1β and 2(CPT1β and CPT2); uncoupling protein 2 (UCP2); acyl-coenzyme A oxidase (AOX), translocase of the inner mitochondrial membrane (TIM23).

To analyze the anti-atherogenic effect of FLS in obese mice, we examined the expression levels of lipoprotein lipase (LPL), the main enzyme involved in the catabolism of atherogenic lipoproteins in the periphery of the body. After overnight fasting, the pool of atherogenic cholesterol is composed of LDL, intermediate density lipoprotein (IDL), and very low density lipoprotein (VLDL), which is the main carrier of TG in the fasting state. LPL-mediated lipolysis of TG-rich lipoproteins releases free fatty acids (FA) that are taken up locally and processed in a tissue-specific manner [30]. Muscle LPL mRNA levels were significantly upregulated in the HFDC group (Figure 4A), suggesting increased VLDL catabolism, which was not accompanied by changes in the very low density lipoprotein receptor (VLDLR) mRNA levels (Figure 4A).

We next analyzed the expression levels of genes involved in FA utilization in muscle. mRNA levels of the peroxisome proliferator-activated receptor α (PPARα), the main transcription factor controlling FA utilization [31] and the PPARγ coactivator-1α, (PGC-1α) [32], remained unaffected upon high-fat feeding (Figure 4B), in agreement with a previous report [33]. However, these mRNA levels were significantly elevated in muscle of HFDC mice (Figure 4B). In agreement, expression levels of PPARα-target genes controlling FA uptake (cluster of differentiation 36 (CD36)) [33], uptake and activation (fatty acid transport protein 1 and 4 (FATP1 and FATP4) [34], and peroxisomal and mitochondrial β-oxidation (acyl-coenzyme A oxidase (AOX)) [35], and carnitine palmitoyltransferases 1β and 2 (CTP1β and CTP2) [36], were significantly elevated in HFDC group (Figure 4C, 4D). In addition, the mRNA expression levels of uncoupling protein 2 (UCP2), which attenuates reactive oxygen species (ROS) production thus protecting against oxidative stress [37], were also elevated (Figure 4D). Note that muscle TG and FA levels were reduced in HFDC compared to HFD group (Table 1 and Figure S3). Furthermore, given the elevation of PGC-1α, a transcriptional regulator of mitochondrial biogenesis [38], [39], we measured by western blot analysis the expression levels of TIM 23 (a subunit of the translocase of the mitochondrial inner membrane), a mitochondrial-specific protein whose levels correlate with mitochondrial number [40]. The levels of TIM23 protein, which were significantly reduced upon high-fat feeding (HFD vs. LFD, P<0.05), were restored to baseline with flavonoid supplementation (HFDC vs. HFD, P<0.01; no difference between LFD and LFDC; Figure 4E and 4F).

Overall, our data suggest that, in muscle of obese mice, FLS increases FA utilization, as a result of increased mitochondrial proliferation, and also increases VLDL catabolism.

### FLS increases AMPK phosphorylation in adiponectin-target tissues

**Figure 5:**
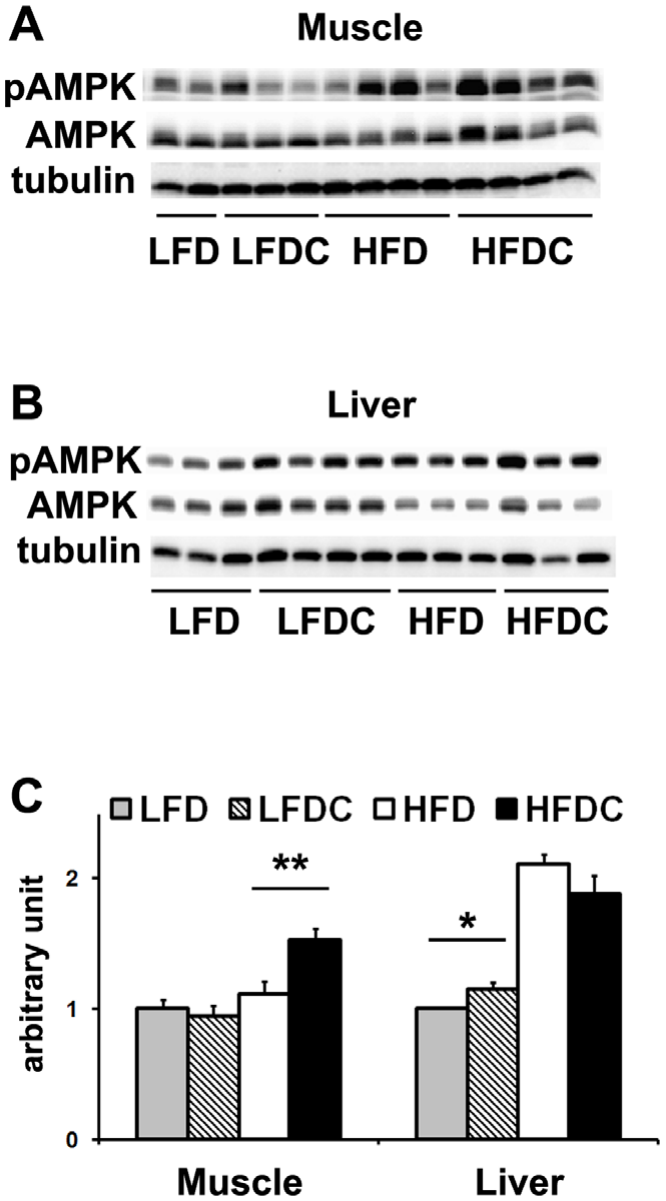
FLS increases AMPK phosphorylation in liver in non-obese mice and in muscle in obese mice. A and B, representative western blots of muscle (A) and liver (B) samples. 70 µg of total protein was subjected to 12.5% SDS-PAGE under reducing, heat-denaturing conditions. C, quantitative analysis of western blots, pAMPK/AMPK ratio. Samples were repeated on different western blot membranes. LFD group was used as a reference for quantification. Data are expressed as mean±SE. *P<0.05, **P<0.01, LFDC vs. LFD or HFDC vs. HFD; n = 3−6 (liver) and n = 8 (muscle) in each group.

The AMP-activated protein kinase (AMPK) promotes mitochondrial biogenesis and FA oxidation in muscle *via* direct upregulation of the expression of PPARα target genes and PGC-1α [41]. In agreement with the results shown above, we found a significant increase in AMPK phosphorylation (phospho-AMPK/AMPK ratio, Figure 5A and 5C) and phospho-AMPK content with no changes in total AMPK content (AMPK/tubulin ratio, data not shown) in muscle of HFDC mice compared to all other groups.

AMPK phosphorylation was increased in liver of LFDC vs. LFD mice (Figure 5B and 5C) though total AMPK was unchanged (data not shown). In contrast, AMPK phosphorylation was not different between HFDC and HFD groups (Figure 5B and 5C). However, high-fat diet increased hepatic AMPK phosphorylation compared to mice on low-fat diet (Figure 5B and 5C). Interestingly, in obese animals the elevated phospho-AMPK signal was accompanied by reduced AMPK content with similar tubulin levels (Figure 5B), suggesting an increased efficiency of AMPK phosphorylation.

In conclusion, we show that the sites of AMPK activation in adiponectin-target tissues upon FLS are different in obese and non-obese mice. AMPK phosphorylation was significantly increased in muscle of HFDC group and in liver of LFDC vs. LFD and obese vs. non-obese mice.

### Effect of FLS on visceral fat mass

**Figure 6:**
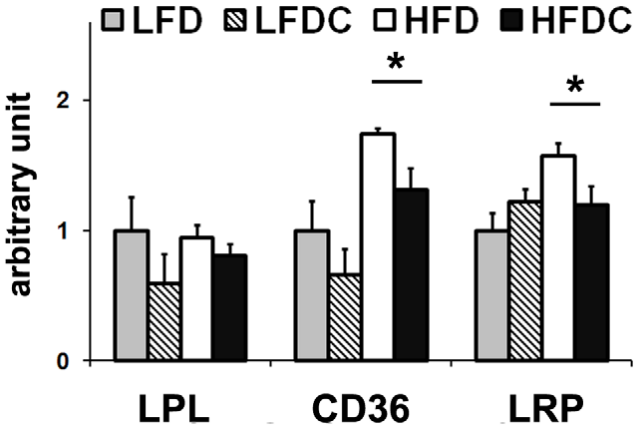
FLS inhibits lipid accumulation in visceral adipose tissue in obese mice. Real-time RT-PCR analysis of genes involved in lipid accumulation: lipolysis (LPL), FFA uptake (CD36) and lipoprotein uptake (LRP). LFD group was used as a reference for quantification. Data are expressed as mean±SE. *P<0.05, HFDC vs. HFD. Lipoprotein related protein (LRP).

To gain insights into the mechanism underlying visceral fat reduction in HFDC group, we measured mRNA levels of genes controlling lipolysis, lipid uptake and utilization in adipose. No changes were observed in LPL mRNA levels in visceral fat (Figure 6), strongly supporting the hypothesis of a muscle specific activation of LPL. mRNA levels of CD36 and LRP were significantly reduced in obese mice upon FLS. No changes were observed upon FLS in mRNA levels of PPARγ, the transcriptional factor primarily responsible for adipocyte differentiation, of genes involved in FA utilization (AOX, CPT1α, CPT2, FATP1, and FATP4), of LDLR, SRBI, and hormone sensitive lipase, the major gene controlling lipolysis in fat tissues [42], regardless of the dietary regimen (data not shown).

Thus, we speculate that reduced lipid uptake is presumably the primary cause of visceral fat reduction observed in HFDC group.

### FLS affects expression levels of adiponectin receptors only in liver

We measured the expression levels of the adiponectin receptors, AdipR1 and AdipR2 in skeletal muscle, liver and visceral adipose. Consistent with current knowledge [8], the expression levels of both AdipR1 and AdipR2 were decreased in adipose tissue upon high-fat feeding. However, no changes were observed upon FLS (Figure S3). No differences were found in muscle for both receptors between any groups (Figure S3). In contrast, hepatic AdipR2 expression levels were increased in LFDC vs. LFD and obese mice vs. non-obese (Figure S2B).

## Discussion

The molecular mechanisms underlying the effects of flavonoids have been poorly understood. Based on chemical structures [43], the health benefits of flavonoids have been generally attributed to their antioxidant and free radical scavenging properties. However, most flavonoids are poorly absorbed, extensively metabolized, and rapidly excreted from the body. Consequently, their concentration in serum and tissues is exceedingly low for the direct antioxidant activity in the periphery [3], but relatively high in the gastrointestinal tract where they can act as local antioxidants and antipathogenic agents [4]. However, tissue concentrations of flavonoids can be sufficient to affect the action of enzymes, receptors and transcription factors, and to act beyond the gastrointestinal tract *in vivo* [3], [4]. Growing evidence from both *in vivo* and *in vitro* studies suggests that polyphenols from food can function synergistically, acting on the same or multiple targets [44].

The consumption of cranberry and cranberry products is associated with multiple benefits to human health. Cranberry is an abundant source of a heterogeneous group of flavonoids, including flavonols (mainly quercetin and myricetin glycosides), anthocyanins, flavan-3-ols (procyanidins and a diverse group of proanthocyanidins with unique structural characteristics [4]), and phenolic acids [45], which differ in bioavailability and function [4].

The original goal of our study was to evaluate the use of dietary supplementation with cranberry flavonoids in amelioration of metabolic abnormalities observed in obese mice, analyze their effects in normal mice and raise possible mechanisms for how these effects might be occurring. FLS indeed decreased serum atherogenic cholesterol in normal mice and improved serum lipid profile and insulin sensitivity status in obese animals. Surprisingly, FLS elevated plasma adiponectin levels in both obese and non-obese mice. Notably, an elevation in serum adiponectin levels upon supplementation with different polyphenols was previously reported [46]–[49]. Among other adipokines, adiponectin is considered an important link between obesity and obesity-related disorders, including atherosclerosis and insulin resistance. In our study, the development of obesity resulted in a decline of adiponectin mRNA levels in visceral fat not accompanied by a reduction in circulating adiponectin protein, but rather by its increase. It is well-described in rodent models of diet-induced obesity that reduction in mRNA levels of adiponectin in visceral adipose tissue does not necessarily lead to the decrease in circulating protein levels presumably due to increase in visceral adipose tissue mass and significant input of other fat deposits [50]–[52].

Adiponectin undergoes extensive post-translational modifications that are extremely important in controlling oligomerization, secretion, functionality, and stability of the protein [26]. Consistent with previous reports [8], we found that high-fat feeding alters the circulating adiponectin profile. However, we also show that these alterations are reversed by FLS. Reduced HMW and increased LMW adiponectin multimers observed in obesity and diabetes [26], [27], [53], [54] have been associated with succination of adiponectin itself [53] and chaperone proteins involved in its maturation and secretion [26], [54]. Oxidative stress in diabetes increases the levels of protein succination [54]. Furthermore, flavonoids elevate the levels of glutathione, the major contributor to the intracellular redox state [55], by regulating the transcription of enzymes involved in its synthesis [55]. Therefore, we speculate that flavonoids can influence the plasma adiponectin profile by affecting the plasma adiponectin profile by influencing the adipocyte redox status altered by high-fat feeding.

In addition to intracellular posttranslational modifications, adiponectin can also be activated in the circulation by proteolytic cleavage [26]. This cleavage occurs at several sites of the N-terminal collagenous domain, releasing truncated forms. The fully truncated protein (18 kDa), globular adiponectin, is unstable and presumably represents the active state of adiponectin in muscle [26], [56]. We hypothesize that the minor adiponectin bands detected by western blot correspond to the initial cleavage products (above 25 kDa). At present, we cannot establish why the levels of truncated protein were decreased upon high-fat feeding but restored to normal in HFDC group. This effect might be due to increased cleavage efficiency or linked to the elevated levels of adiponectin.

The action of adiponectin in its target tissues is mediated by its binding to specific receptors [8]. AdipR1, a high affinity receptor for globular adiponectin, is abundantly expressed in skeletal muscle and liver, whereas AdipR2, predominantly expressed in liver, binds both full-length and globular adiponectin with lower affinity [8]. Since no differences between any groups were found in muscle for both receptors, we assume that the availability of the ligand, globular adiponectin (that is present in the circulation at extremely low levels), rather than the expression level of receptors is the limiting factor that determines adiponectin signaling in muscle. In contrast, given 1. that the expression profile of hepatic AdipR2 matches the profile of AMPK activation; 2. the abundance of full-length adiponectin, the main active adiponectin form in liver; and 3. the lower affinity of AdipR2 for its ligand, it is likely that AdipR2 expression level rather than its ligand availability determines adiponectin signaling in the liver.

AMPK, the main enzyme controlling cellular energy metabolism, is a major downstream target of adiponectin [57]. Once activated, AMPK switches off ATP-consuming anabolic processes and switches on ATP-generating catabolic pathways [58]. Thus, adiponectin signaling mediated by AMPK is known to upregulate mitochondrial biogenesis and FA utilization in skeletal muscle and downregulate gluconeogenesis and sterol synthesis in liver [58]. In our experimental model, AMPK was activated in the main adiponectin target tissues, depending upon the metabolic status. FLS activated AMPK in muscle of obese mice, resulting in elevation of expression levels of PPARα target genes, including LPL and those controlling FA utilization. Activation of PPARα target genes is a well-documented effect of adiponectin in muscle [8], [59]. Moreover, enhanced VLDL catabolism *via* activation of muscle LPL has been demonstrated upon acute adiponectin treatment *in vivo* [21]. We therefore hypothesize that adiponectin upregulates LPL expression through PPARα activation, in turn mediated by AMPK. However, the link between adiponectin-AMPK and PPARα remains unclear. We propose that adiponectin enhances PPARα activity *via* AMPK through activation of PGC-1α and PPARα itself, and through the enhanced production of its ligands. Adiponectin has been shown to elevate FA oxidation *in vitro* by sequential activation of AMPK, p38 MAPK, and PPARα [59]. Also, PGC-1α is a PPARα transcriptional coactivator of genes encoding mitochondrial FA β-oxidation [32] and one of the primary targets of AMPK [60]. Furthermore, PPARα is a ligand-induced transcription factor [61] and FAs released during the LPL-enhanced lipolysis of VLDL ensure additional positive feedback on PPARα activity [61]. Phosphorylation of PPARα also markedly increases coactivation by PGC-1α and significantly enhances ligand-dependent transactivation [62]. In addition, activated PPARα and PGC-1α regulate their own expression [60], [63], [64]. Finally, it has recently been shown that, in muscle, the binding of adiponectin to AdipR1 increases PGC-1α expression and activity through signaling cascades involving AMPK activation [64].

Plasma TG concentrations are inversely correlated with adiponectin levels in humans and animal models [8]. In our experiments, the difference in fasting serum TG levels among groups did not reach statistical significance likely due to considerable inter-individual variability. However, even drastic elevation of adiponectin levels only results in a moderate reduction in fasting TG levels (40%) [21], comparable to that observed in our study. Hydrolysis of TG in TG-rich lipoproteins is mediated by LPL, a key enzyme in lipoprotein metabolism [30]. Upregulation of LPL activity in muscle also stabilizes HDL fraction as remnant particles released during hydrolysis of TG-rich lipoproteins contributing to the maturation of HDL precursors [65]. Indeed, plasma adiponectin levels are positively correlated with levels of HDL and HDL cholesterol [66]–[68] and negatively correlated with apolipoprotein A–I clearance from the circulation and with the levels of atherogenic large VLDL and small LDL [67]. Therefore, we propose that 1. an increase of muscle LPL expression upon PPARα elevation resulted in accelerated VLDL catabolism and consequently facilitated FA utilization in muscle; 2. an elevation of VLDL catabolism could be primarily responsible for the significant reduction of atherogenic cholesterol and increase of percentage of HDL cholesterol observed upon FLS in obese mice.

Adiponectin is known to downregulate hepatic gluconeogenesis and improve insulin sensitivity in skeletal muscle [57] by several mechanisms activated *via* adiponectin/AdipR1 signaling [64]. Muscle lipid content is one of the crucial factors determining insulin sensitivity. Indeed, in HFDC group (compared to HFD mice), activation of AMPK pathway in skeletal muscle resulted in a significant decrease in muscle lipid content and improved insulin sensitivity, as indicated by the fasting insulin levels and the HOMA index. In our model, we did not observe significant changes in fasting glucose levels despite the increased insulin resistance observed in HFD mice. However, this finding was not surprising. Indeed, it is known that in mice, an overnight fast (as performed in our study) significantly suppresses basal plasma glucose levels such that there are no differences between high-fat diet and chow-fed animals. This is mainly due to the fact that mice are nocturnal feeders, with about 70% of their daily caloric intake occurring during the dark cycle, and their metabolic rate is much higher than in humans [69]. Moreover, we found a trend towards a decrease in hepatic G6P mRNA levels in LFDC vs. LFD group (−32% LFDC vs. LFD, p = 0.06), where phosphorylation of AMPK was elevated. The lack of statistical significance might result from compensatory mechanisms maintaining homeostatic glucose levels during prolonged fasting periods [70].

**Figure 7:**
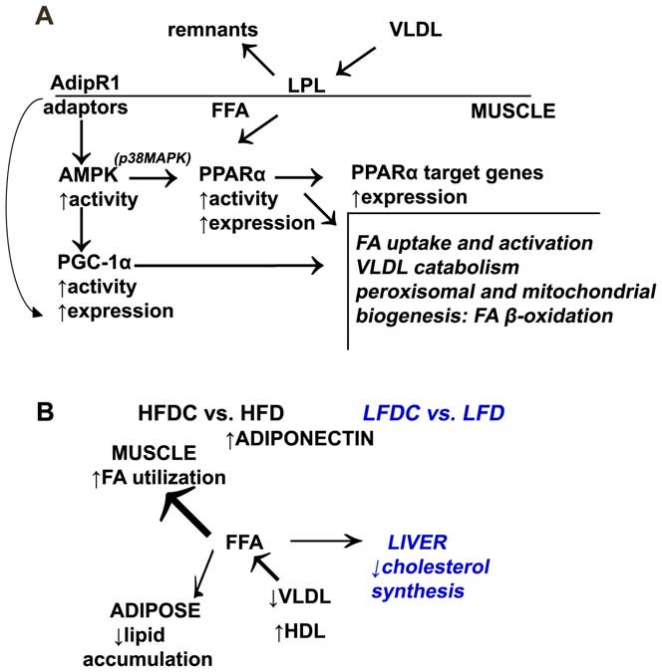
Molecular mechanisms underlying the anti-atherogenic effects of flavonoids in normal and obese mice. A, activation of AMPK pathway in muscle of HFDC mice. Adiponectin/AdipR1 signaling in muscle activates the AMPK pathway, resulting in elevated transcriptional activity of PGC-1α and PPARα, and consequently increased expression of their target genes. B, molecular mechanisms underlying the anti-atherogenic effects of flavonoids are different in normal and obese mice.

In summary (Figure 7A), we propose that, adiponectin/AdipR1 signaling in muscle activates the AMPK pathway, resulting in elevated transcriptional activity of PGC-1α and PPARα, and consequently increased expression of their target genes, which improves the plasma cholesterol profile and insulin sensitivity in HFDC group. We cannot exclude that, in addition to the PGC-1α/PPARα pathway, AMPK activation could also regulate FA oxidation *via* ACC phosphorylation [8]. Overall, our data are in agreement with recently published *in vivo* and *in vitro* [64], [71] studies showing that adiponectin signaling regulates mitochondrial biogenesis and oxidative stress in skeletal muscle.

We cannot rule out that resistin, downregulated upon FLS, is an additional player of flavonoid action. Indeed, resistin has been shown to inhibit AMPK phosphorylation and FA utilization in muscle [72]. However, unlike adiponectin, resistin affects FA uptake *via* CD36 translocation but not its expression level [72]. Moreover, resistin has not been correlated with VLDL catabolism [73]. Therefore, we are inclined to hypothesize that resistin is not the primary effector of the flavonoid action in our model.

Finally, we speculate that, downregulation of cholesterol synthesis in the liver, which is the major site of total body cholesterol synthesis in rodents [74], significantly reduced plasma atherogenic cholesterol levels in LFDC mice. Activation of AMPK in liver has been shown to inhibit HMG-R, the rate-limiting enzyme of cholesterol synthesis *via* its phosphorylation [75]. In our study, high-fat feeding and FLS of normal mice resulted in elevation of AMPK phosphorylation in the liver and reduced expression not only of HMG-R but also of other key genes of the cholesterol biosynthetic pathway, such as SS, FDS, and HMG-S. In agreement with the current notion that excess cholesterol coordinately regulates expression of all endoplasmic reticulum genes involved in cholesterol synthesis pathway, including HMG-R [29], these data support our interpretation that cholesterol synthesis is downregulated in LFDC mice. Further studies will be needed to unequivocally confirm this hypothesis. Sharp, switch-like control of the SREBP pathway maintains hepatic cholesterol content in a very narrow range by regulating its synthesis and uptake [29]. Indeed, in our study, hepatic cholesterol levels did not change regardless of the dietary regimen. Wild-type mice adapt quickly to cholesterol rich diets and are relatively resistant to the development of arthrosclerosis due to high level of HDL and low level of atherogenic lipoproteins compared to humans [76], [77]. Moreover, due to compensatory increase in bile synthesis [77] and downregulation of cholesterol synthesis [77], [78], wild-type mice maintain relatively low hepatic cholesterol levels. Although we did not enrich diet with cholesterol, mice maintained on high-fat diet consumed more cholesterol from the food (Table S2) and showed downregulation of the genes of the hepatic cholesterol synthesis. Downregulation of the SREBP pathway in transgenic mice also reduces plasma total cholesterol despite the reduction of hepatic LDL clearance, mainly due to the decrease in HDL levels [79]. We did not expect that the observed 30% reduction in hepatic LDLR expression level (LFDC vs. LFD group) would have a significant effect of serum cholesterol levels. Indeed, LDLR is expressed in liver as well as in peripheral tissues. In our study LDLR expression levels were similar in muscle of all groups and showed a tendency to increase in adipose of normal mice upon FLS (+80%, LFDC vs. LFD, p = 0.08, data not shown). Moreover, LDL receptor is a key component of the feedback regulatory mechanisms that maintain constant levels of cholesterol within the cell, while the circulating levels in the form of lipoproteins can largely fluctuate [80].

We cannot exclude that hepatic cholesterol uptake was elevated in the fed state in the LFDC group. This would ultimately increase hepatic cholesterol content and subsequently inhibit the SREBP pathway [29]. Notably, LDLR has been shown to be upregulated by supplementation with different flavonoids [81], [82].

In the LDFC, hepatic mRNA levels of SREBP1c, the main transcription factor involved in the regulation of FA synthesis [31], as well as those of fatty acid synthase (FAS) were also downregulated (Figure S4). However, the upregulation of the stearoyl-coenzyme A desaturase (SCD) mRNA levels in this group suggests the existence of more complex regulatory effects of flavonoids on hepatic fatty acid synthesis that warrant further studies.

Despite the reduction in visceral fat mass and increased FA utilization observed in HFDC group, we did not observe changes in total adiposity. In fact, muscle TG content and net loss of visceral adipose tissue are extremely low compared to the total body fat mass to make this difference remarkable. Adipogenesis does not seem to be changed in our experimental model, likely because PPARγ, as well as genes involved in lipolysis and FA β-oxidation were not affected by FLS. Therefore our data suggest that the reduction of visceral fat mass is mainly due to decreased lipid uptake by adipose tissue as a result of increase of FA utilization in skeletal muscle. We propose that activation of LPL in muscle upon FLS makes this tissue to act as a “sink” for circulating FA, thus reducing their availability for storage in adipose, the only tissue that stores FA under normal physiological conditions. We also cannot exclude adipose redistribution among different fat depots in the body. Notably, both loss of visceral fat and redistribution of fat from visceral into subcutaneous areas are considered beneficial effects [83].

Overall our data demonstrate that flavonoids affect different molecular pathways in normal *versus* pathological conditions (Figure 7B). Further research is still required to establish the primary targets of flavonoid actions in the body and the direct link between flavonoids and the molecular pathways that we have shown to be affected. We speculate that flavonoids could activate internal antioxidant pathways that drastically change adiponectin assembling in adipocytes and trigger the adiponectin pathway. We believe that the adiponectin-driven metabolic response is one of the previously un-recognized mechanisms of flavonoid action *in vivo*, since several studies reported elevation of adiponectin levels or improvement of mitochondrial biogenesis or similar changes in cholesterol metabolism under supplementation with different polyphenols [46], [47]–[49], [82], [84]–[86].

## Supporting Information

Figure S1 **Glucose Tolerance Test (GTT) in mice at 14 weeks of age.** GTT in mice at 14 wks of age. Glucose (1.5 g/ kg body weight) was injected intraperitoneally after overnight fasting (16 hours). Plasma glucose was measured using OneTouch Ultra Glucose Meter (LifeScan) at 0, 15, 30, 60, and 90 min after injection. n = 3−4 animal per group. Data are expressed as means±SE.
(TIF)

Figure S2 **Muscle lipids in mice at 24 weeks of age.** Total lipids from liver and muscle samples were extracted according to the Folch extraction protocol [17]. The extracts dissolved in chloroform with 2% Triton X100 were evaporated under nitrogen and dissolved in water followed by measurement of FA using the HR Series NEFA-HR(2) kit (Wako Chemicals, Richmond, VA, USA). LFD group was used as a reference for quantification. ^#^P<0.001 LFDC vs. LFD and HFDC vs. HFD. n = 8 animal per group. Data are expressed as means±SE.
(TIF)

Figure S3 **FLS increases expression levels of hepatic AdipoR2 in non-obese mice.** Real-time RT-PCR analysis of adiponectin receptors AdipoR1 (A) and AdipoR2 (B) in different tissues. LFD group was used as a reference for quantification. Data are expressed as mean±SE. *P<0.05, LFDC vs. LFD n = 8 in each group.
(DOCX)

Figure S4 **FLS affects expression of genes involved in FA synthesis in the liver of normal mice.** Real-time RT-PCR analysis of genes involved in FA synthesis in the liver. LFD group was used as a reference for quantification. Data are expressed as mean±SE. *P<0.05, LFDC vs. LFD n = 8 in each group.
(DOCX)

Table S1 **Flavonoid composition of cranberry extract.**
(DOCX)

Table S2 **Diet composition.**
(DOCX)

Table S3 **Primer sequences for quantitative real time PCR.**
(DOCX)

Table S4 **Body composition, tissue weight, and fasting serum parameters of mice at 14 weeks of age.**
(DOCX)
