# Supplementary figures and images for: Insights into the Molecular Mechanisms of the Anti-Atherogenic Actions of Flavonoids in Normal and Obese Mice
Source: PLoS One. 2011 Oct 10;6(10):e24634. doi: 10.1371/journal.pone.0024634 (PMC3189911; doi:10.1371/journal.pone.0024634)

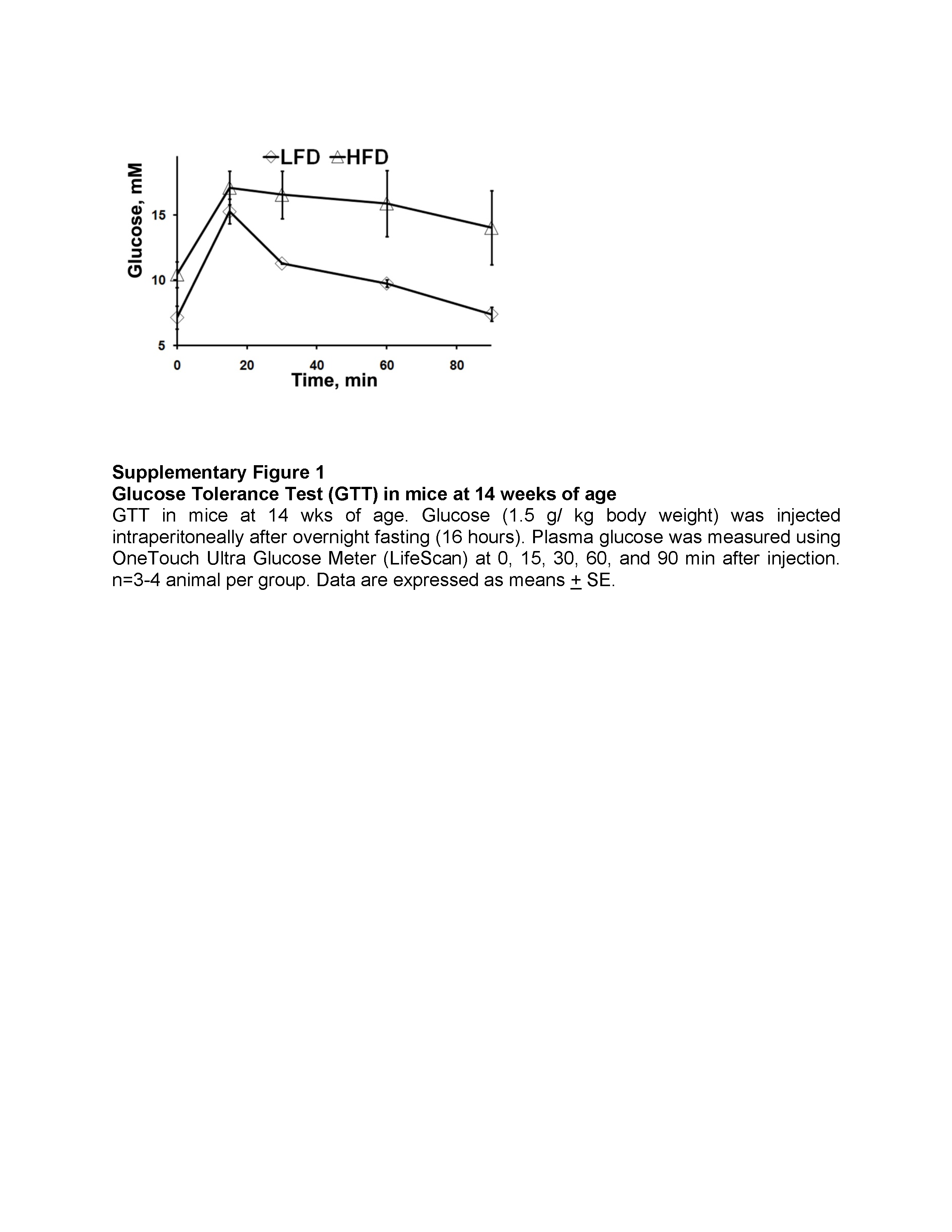

Supplement: Figure S1 — Glucose Tolerance Test (GTT) in mice at 14 weeks of age. GTT in mice at 14 wks of age. Glucose (1.5 g/ kg body weight) was injected intraperitoneally after overnight fasting (16 hours). Plasma glucose was measured using OneTouch Ultra Glucose Meter (LifeScan) at 0, 15, 30, 60, and 90 min after injection. n = 3−4 animal per group. Data are expressed as means±SE. (TIF) [file pone.0024634.s001.tif]

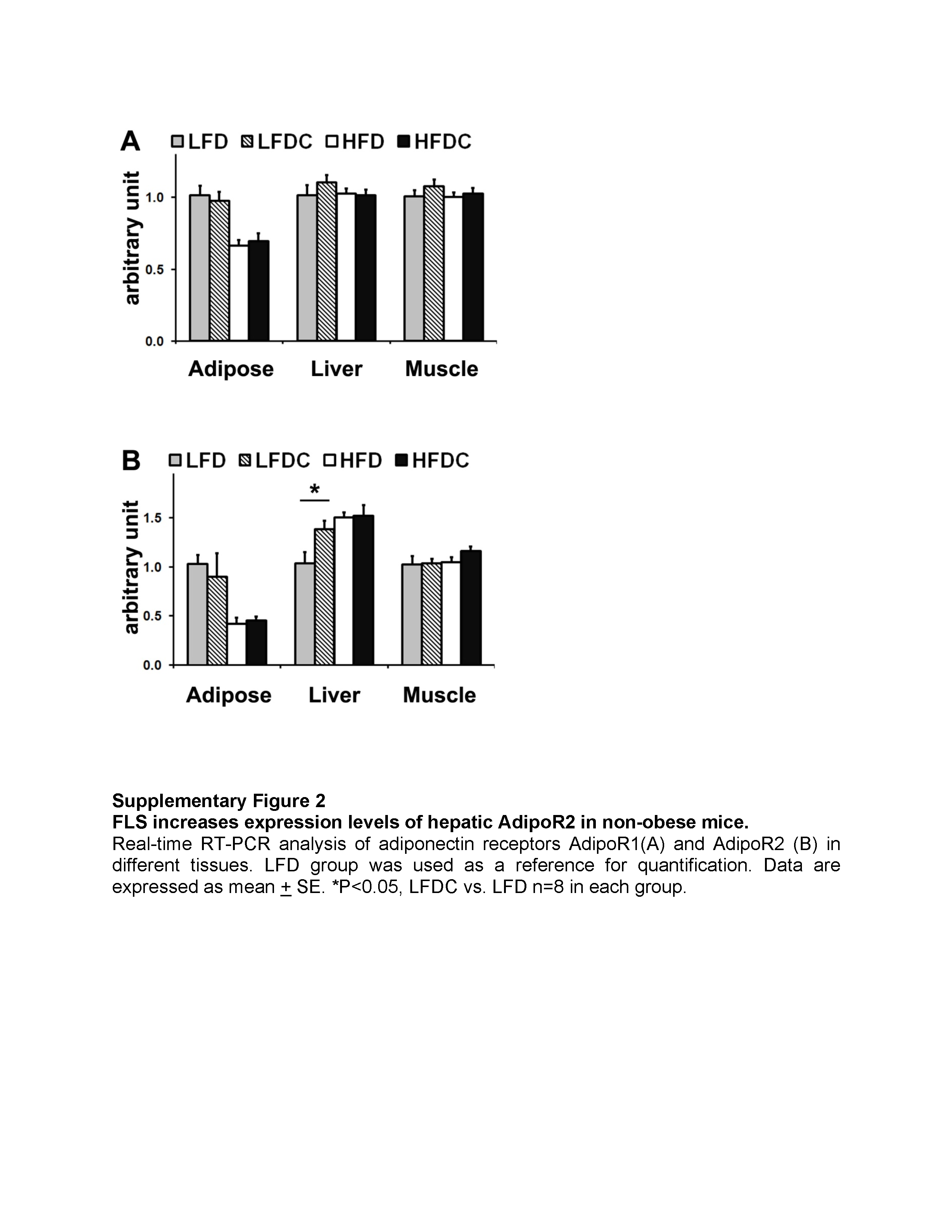

Supplement: Figure S2 — Muscle lipids in mice at 24 weeks of age. Total lipids from liver and muscle samples were extracted according to the Folch extraction protocol [17]. The extracts dissolved in chloroform with 2% Triton X100 were evaporated under nitrogen and dissolved in water followed by measurement of FA using the HR Series NEFA-HR(2) kit (Wako Chemicals, Richmond, VA, USA). LFD group was used as a reference for quantification. #P<0.001 LFDC vs. LFD and HFDC vs. HFD. n = 8 animal per group. Data are expressed as means±SE. (TIF) [file pone.0024634.s002.tif]

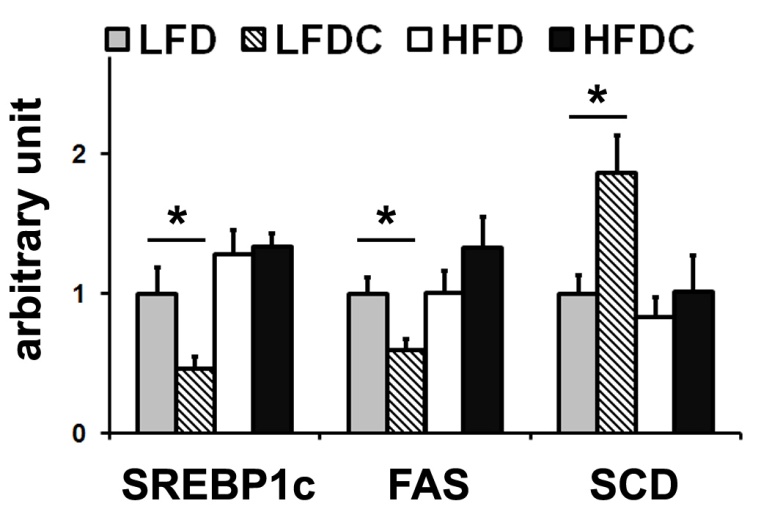


**Supplementary Figure 3**

Supplement: Figure S3 — FLS increases expression levels of hepatic AdipoR2 in non-obese mice. Real-time RT-PCR analysis of adiponectin receptors AdipoR1 (A) and AdipoR2 (B) in different tissues. LFD group was used as a reference for quantification. Data are expressed as mean±SE. *P<0.05, LFDC vs. LFD n = 8 in each group. (DOCX) [file pone.0024634.s003.docx]

**
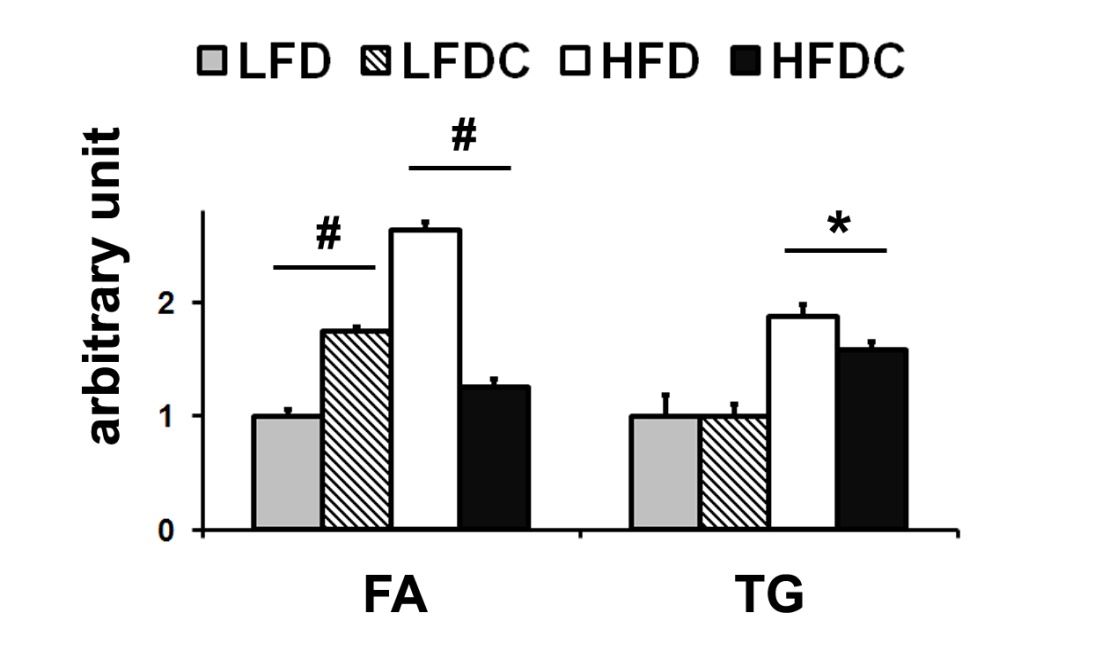
**

**Supplementary Figure 4**

Supplement: Figure S4 — FLS affects expression of genes involved in FA synthesis in the liver of normal mice. Real-time RT-PCR analysis of genes involved in FA synthesis in the liver. LFD group was used as a reference for quantification. Data are expressed as mean±SE. *P<0.05, LFDC vs. LFD n = 8 in each group. (DOCX) [file pone.0024634.s004.docx]
