# Supplementary material for: Insights into the Molecular Mechanisms of the Anti-Atherogenic Actions of Flavonoids in Normal and Obese Mice
Source: PLoS One. 2011 Oct 10;6(10):e24634. doi: 10.1371/journal.pone.0024634 (PMC3189911; doi:10.1371/journal.pone.0024634)
Supplement: Table S1 — Flavonoid composition of cranberry extract. (DOCX) [file pone.0024634.s005.docx]

**Supplementary Table 1. Flavonoid composition of cranberry extract**

| Flavonoids extract, mass % in diet | 2 |
| --- | --- |
| PAC, mass % in extract | 9 |
| PAC, mass % in diet | 0.18 |
| Anthocyanins, mass % in extract | 5 |
| Anthocyanins, mass % in diet | 0.01 |
| Flavonols, mass % in extract | 1.4 |
| Flavonols, mass % in diet | 0.028 |
| Flavonols composition, mole% | |
| Merycetin-3-galactoside | 7.8 |
| Quercetin-3-galactoside | 28.6 |
| Quercetin-3-glucoside | 1.1 |
| Quercetin-3-arabinopyranoside | 5.2 |
| Quercetin-3-arabinofuranoside | 4.1 |
| Quercetin-3-rhamnoside | 17.2 |
| Quercetin | 36.1 |
