# Supplementary material for: Insights into the Molecular Mechanisms of the Anti-Atherogenic Actions of Flavonoids in Normal and Obese Mice
Source: PLoS One. 2011 Oct 10;6(10):e24634. doi: 10.1371/journal.pone.0024634 (PMC3189911; doi:10.1371/journal.pone.0024634)
Supplement: Table S2 — Diet composition. (DOCX) [file pone.0024634.s006.docx]

**Supplementary Table 2. Diet composition**

|  | HFD | LFD |
| --- | --- | --- |
| Fat (lard), % | 34.9 | 4.3 |
| Cholesterol, ppm | 301 | 18 |
| Fiber (max), % | 6.5 | 4.7 |
| Carbohydrates, % | 6.5 | 67.4 |
| Vitamin A, IU/g | 25 | 25 |
| Energy, (kcal/g) | 5.15 | 3.78 |
| from |  |  |
| Protein | 18.4 | 18.3 |
| Fat | 61.3 | 10.2 |
| Carbohydrates | 20.2 | 71.5 |
