# Supplementary material for: Insights into the Molecular Mechanisms of the Anti-Atherogenic Actions of Flavonoids in Normal and Obese Mice
Source: PLoS One. 2011 Oct 10;6(10):e24634. doi: 10.1371/journal.pone.0024634 (PMC3189911; doi:10.1371/journal.pone.0024634)
Supplement: Table S3 — Primer sequences for quantitative real time PCR. (DOCX) [file pone.0024634.s007.docx]

**Supplementary Table 3. Primer sequences for quantitative real time PCR**

| Gene | Forward primer 5’→3’ | Reverse primer 5’→3’ |
| --- | --- | --- |
| Lipolysis | | |
| LPL | CCCTAAGGACCCCTGAAGAC | GGCCCGATACAACCAGTCTA |
| Lipoprotein uptake | | |
| LDLR | AGGCTGTGGGCTCCATAGG | TGCGGTCCAGGGTCATCT |
| VLDLR | AGCACCACAGATCAATGACC | CTCTCGTCCATTTTCTTCGAGA |
| LRP | ACAAGGACTGTACCGATGGC | GGGTACTCACACTCAGGGGA |
| FA utilization | | |
| PPARα | CAGCAACAACCCGCCTTTT | GCAGTGGAAGAATCGGACCTC |
| PGC-1α | GTAAATCTGCGGGATGATGG | AGCAGGGTCAAAATCGTCTG |
| CD36 | CAGTCGGAGACATGCT | CTCGGGGTCCTGAGTT |
| FATP1 | TGCTTTGGTTTCTGGGACTT | GCTCTAGCCGAACACGAATC |
| FATP4 | GATGGCCTCAGCTATCTGTGA | GGTGCCCGATGTGTAGATGTA |
| CPT1β | TGCCTTTACATCGTCTCCAA | AGACCCCGTAGCCATCATC |
| CPT2 | GCCCAGCTTCCATCTTTACT | CAGGATGTTGTGGTTTATCCGC |
| UCP2 | CAGAGCACTGTCGAAGCCTA | GTATCTTTGATGAGGTCATA |
| AOX | TCAACAGCCCAACTGTGACTTCCATTA | TCAGGTAGCCATTATCCATCTCTTCA |
| Cholesterol and FA synthesis | | |
| SREBP1c | GGAGCCATGGATTGCACATT | GGCCCGGGAAGTCACTGT |
| SREBP2 | CAAGTCTGGCGTTCTGAGGAA | ATGTTCTCCTGGCGCAGCT |
| FAS | GCTGCGGAAACTTCAGGAAAT | AGAGACGTGTCACTCCTGGACTT |
| SCD | CCGGAGACCCCTTAGATCGA | TAGCCTGTAAAAGATTTCTGCAAACC |
| HMG-R | CTTGTGGAATGCCTTGTGATTG | AGCCGAAGCAGCACATGAT |
| HMG-S | GCCGTGAACTGGGTCGAA | GCATATATAGCAATGTCTCCTGCAA |
| FDS | ATGGAGATGGGCGAGTTCTTC | CCGACCTTTCCCGTCACA |
| SS | CCAACTCAATGGGTCTGTTCCT | TGGCTTAGCAAAGTCTTCCAACT |
| Adiponectin | | |
| Adiponectin | AGGCCGTGATGGCAGAGATG | CTTCTCCAGGCTCTCCTTTCCTGC |
| AdipR1 | AAGCACCGGCAGACAAGAGC | AGGAAGAACCAGCCCATCTG |
| AdipR2 | CTGTGTGCTGGGCATTGCAG | AGCCTATCTGCCCTATGGTG |
| Control | | |
| Cyclophilin | ATGTGCCAGGGTGGTGACTT | GCCATCCAGCCATTCAGTCT |
| β-actin | AACACAGTGCTGTCTGGTGG | GAAAGGGTGTAAAACGCAGC |
