# Supplementary material for: Insights into the Molecular Mechanisms of the Anti-Atherogenic Actions of Flavonoids in Normal and Obese Mice
Source: PLoS One. 2011 Oct 10;6(10):e24634. doi: 10.1371/journal.pone.0024634 (PMC3189911; doi:10.1371/journal.pone.0024634)
Supplement: Table S4 — Body composition, tissue weight, and fasting serum parameters of mice at 14 weeks of age. (DOCX) [file pone.0024634.s008.docx]

**Table 4** Body composition, tissue weight, and fasting serum parameters of mice at 14 weeks of age

|  | LFD | HFD |
| --- | --- | --- |
| Body composition | | |
| Body weight, g | 25 + 2 | 39 + 4* |
| Lean mass, g | 19 + 1 | 22 + 1 |
| Total Fat, % | 23 + 3 | 45 + 3* |
| Bone mineral density, mg/cm^2^ | 50.9 + 0.5 | 49 + 1 |
| Tissue weights | | |
| Visceral fat, g | 0.6 + 0.2 | 2.1 + 0.2* |
| Visceral fat, % | 2.4 + 0.5 | 5.4 + 0.3* |
| Mesenteric fat, g | 0.13 + 0.08 | 0.7 + 0.2 |
| Liver, g | 0.9 + 0.1 | 1.3 + 0.2 |
| Fasting serum parameters: Glucose metabolism | | |
| Glucose, mM | 7 + 1 | 10 + 1 |
| GTT, glucose at 90 min, mM | 7 + 1 | 14 + 3 |
| Insulin, ng/mL | 0.6 + 0.3 | 4 + 2 |
| Fasting serum parameters: Lipids | | |
| TG, mg/dL | 52 + 14 | 67 + 11 |
| NEFA, mmol/L | 0.9 + 0.1 | 0.9 + 0.14 |
| T-C, mg/dL | 123 + 5 | 174 + 2^#^ |
| HDL-C, mg/dL | 81 + 5 | 95 + 3* |
| (T-C) – (HDL-C), mg/dL | 41 + 3 | 79 + 2^#^ |
| HDL-C/T-C, % | 66 + 2 | 55 + 1** |
| Fasting serum parameters: Adipokines | | |
| Leptin, ng/mL | 22 + 10 | 143 + 47 |
| Resistin, ng/mL | 32 + 13 | 23 + 3 |
| Adiponectin, µg/mL | 67 + 3 | 60 + 5 |

*P<0.05, **P<0.01, ^#^P<0.001, HFD vs. LFD, n=3-4 animals per group.

Data are expressed as mean + SE
